# Osthole inhibits gastric cancer cell proliferation through regulation of PI3K/AKT

**DOI:** 10.1371/journal.pone.0193449

**Published:** 2018-03-28

**Authors:** Xiaojun Xu, Xiaoyuan Liu, Yan Zhang

**Affiliations:** 1 Department of pharmacy, the Second People's Hospital of Wuxi, Wuxi, P.R. China; 2 Department of pharmacy, the Forth People's Hospital of Wuxi, Wuxi, P.R. China; 3 Department of pharmacy, Wuxi M and Child Health Hospital, Wuxi, P.R. China; Duke University School of Medicine, UNITED STATES

## Abstract

Osthole is an active compound isolated from Chinese herb *Cnidium monnieri* (L.) Cusson, and had been reported to possess antitumor effect. However, the effect of osthole on the gastric cancer cells has not been investigated. In this study, the effects of osthole on the proliferation of human gastric cancer cells were tested. The data showed that osthole treatment significantly inhibited the proliferation of gastric cancer cells and resulted in the cell cycle arrest at G2/M phase in a dose-dependent manner. Western-blot study showed that the expression of cyclin B1 and cdc2 was markedly reduced by osthole. Moreover, expression of PI3K and pAKT was also significantly suppressed, and the results indicated that the inhibition of pAKT, cyclin B1, and cdc2 levels by osthole was notably enhanced by a PI3K inhibitor. These results demonstrate that osthole could inhibit gastric cancer cells proliferation via induction of cell cycle arrest at G2/M phase by the reduction of PI3K/AKT.

## Introduction

Gastric cancer is one of the most aggressive malignancies in the world, especially in China [1,2]. Many progress has been made in the research of gastric cancer, however, surgical resection has remained the best treatment for gastric cancer still now. Besides, locoregional recurrence easily occurs even after complete surgical resection [3]. Therefore, it is imperative to develop novel effective chemotherapeutic drugs for the therapy of gastric cancer.

Osthole, 7-methoxy-8-(3-methyl-2-butenyl)-2H-1-benzopyran-2-one, first isolated from *Cnidium* plant, is highly enriched in mature fruit of *Cnidium monnieri* (Fructus Cnidii) [4,5]. Previous experimental data have revealed that osthole exerts a variety of biological and pharmacological activities including osteogenesis [6], immunomodulation [7], neuroprote ction [8] and antioxidant functions [9], making it a potential functional food and drug candidate. In recent years, accumulating studies have demonstrated that osthole possesses anti-cancer property in various kinds of cancers such as ovarian cancer [10], lung cancer [11,12], sarcoma [13], glioma [14], leukemia [15], hepatocellular carcinoma [16], breast cancer [17] and so on. However, the influence of osthole on the growth of gastric cancer has not been clarified yet. Therefore, the purpose of our study was to explore the effect of osthole on the cell growth and cell cycle of gastric cancer cells and investigate the possible molecular mechanisms involved, in order to clarify the biological and therapeutic functions of osthole-treated gastric carcinoma cells.

## Materials and methods

### Reagents

Osthole was provided by Green Fount Natural Product Co., Ltd. (Xi'an, China) with a purity of ≥98%. RPMI-1640 and trypsin were purchased from Biological Industries (Kibutz Beit Haemek, Israel). Fetal bovine serum (FBS) was purchased from Solarbio Science&Technology (Beijing, China). 3-(4, 5-dimethyl thiazol-2yl)-2, 5-diphenyltetrazolium bromide (MTT), dimethyl sulfoxide (DMSO) and propidium iodide (PI) were obtained from Sigma-Aldrich (St. Louis, USA). Annexin V-PI apoptosis reagents were from Bytime (Shanghai, China). Antibodies were purchased from Santa Cruz Biotechnology (Santa Cruz, CA).

### Cell culture and cell morphology determination

Human gastric cancer cells SGC-7901 and HGC-27 were obtained from the Shanghai cell bank of Chinese academy of sciences (Shanghai, China) and kept in our laboratory. The cells were cultured in RPMI 1640 (Gibco, Invitrogen Corporation, USA) supplemented with 10% FBS, and maintained in a humidified atmosphere with 5% CO_2_ at 37°C. Osthole was dissolved in dimethylsulfoxide (DMSO) at a stock solution of 200 mM. Cells were treated with osthole at final doses of 0–320 μM in culture medium with 10% FBS. After the cells were incubated with osthole for 48 h, cell morphology was measured using a phase contrast microscope.

### Cell viability assay

Cell viability was tested by MTT assay. After treatment with osthole for 48 h, cell viability was assessed by incubation with 20 μL of 5 mg/ml MTT for 2 h at 37°C. Medium with MTT was removed and 150 μL of DMSO was added. The plate was shaken for 10 min until crystals were dissolved and measured at 490 nm by an enzyme-linked immunosorbent assay reader (Bio-RAD, USA).

### Cell cycle assay

Cell cycle analysis was conducted by flow cytometry as described previously [18,19]. The cells were treated with various doses of osthole for 48 h, harvested and fixed in 70% ethanol at 4°C. After 48 h, the cells were rinsed with PBS, incubated with RNase (50 μg/ml), and stained with PI (100 μg/ml) in the dark for 30 min. The cell phase distribution was then tested using a FACScan flow cytometer (Becton Dickinson, San Jose, CA).

### Cell apoptosis assay

Cell apoptosis detection was performed using Annexin V-PI staining and flow cytometry analysis. The cells were treated with various doses of osthole for 48 h, harvested and re-suspended in binding buffer. Then the cells were incubated with Annexin V solution, and stained with PI solution in the dark for 15 min. The cell apoptosis was then detected using a FACScan flow cytometer (Becton Dickinson, San Jose, CA).

### Western blot assay

The cells were harvested, wash with PBS and lysed (lysis buffer: 10 mmol/L Tris-HCl (pH 7.4), 150 mmol/L NaCl, 1% Triton X-100, 1% sodium deoxycholate, 0.1% SDS, 5 mmol/L edetic acid, 1 mmol/L phenylmethysulfonyl fluoride (PMSF), 0.28 kU/L aprotinin, 50 mg/L leupeptin, 1 mmol/L benzamidine, and 7 mg/L pepstatin A). The protein concentration was measured using a bicinchoninic acid (BCA) kit. Proteins were separated by SDS-PAGE and transferred onto nitrocellulose membranes. The membranes were blocked with 5% skimmed milk in Tris buffered saline (TBS) containing 0.1% Tween 20, and incubated with primary antibodies overnight at 4°C. The membranes were washed and incubated with fluorescent secondary antibody for 1 h. The ratio of the protein interested was subjected to GAPDH and was densitometrically analyzed by Odyssey infrared imaging system [4].

### Statistical analysis

Data were expressed as mean ± SD. For statistical analysis, one-way analysis of variance (ANOVA) was performed for comparisons among groups using SPSS 13.0 software. A two-side P value <0.05 was considered statistically significant.

## Results

### Osthole inhibits the cell proliferation of gastric cancer cells

**Fig 1:**
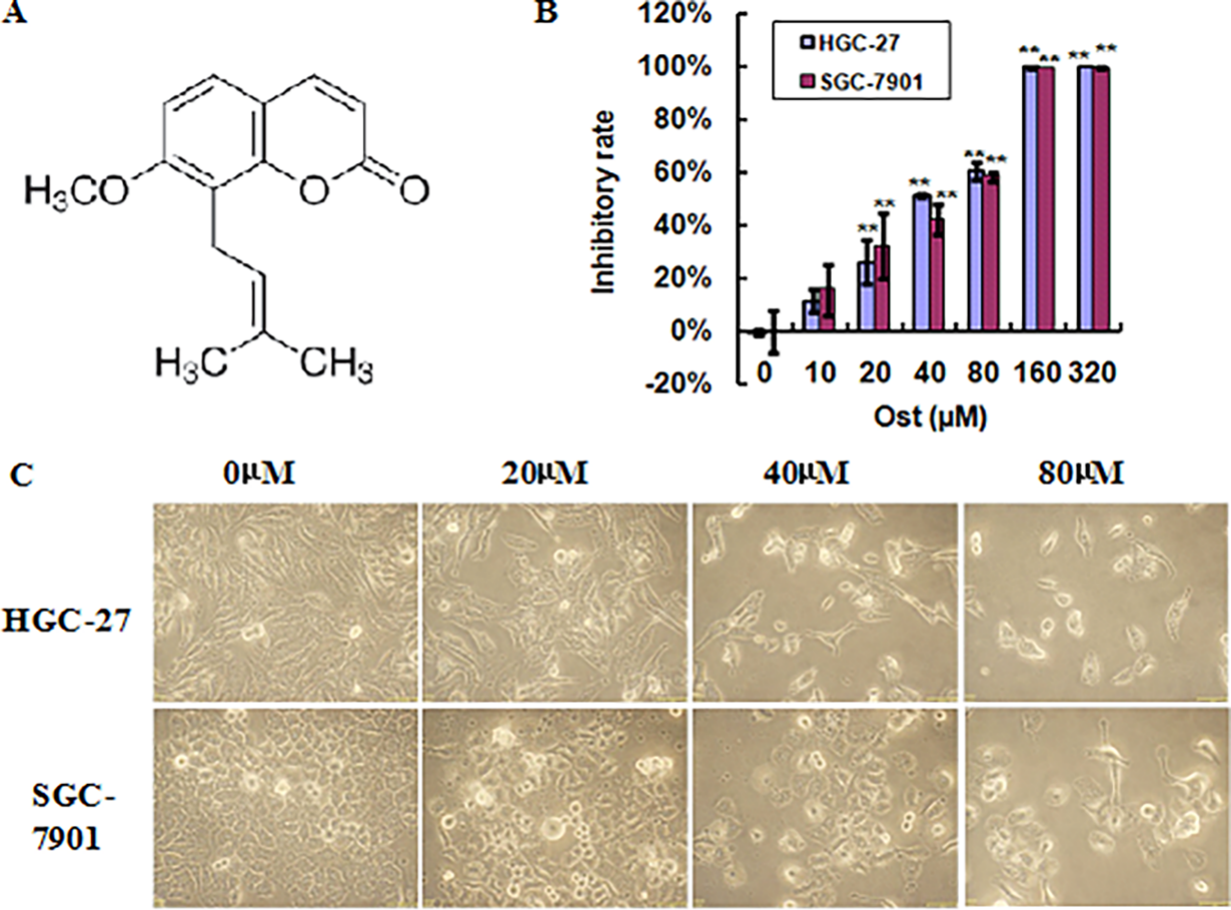
Osthole inhibits the cell growth of gastric cancer cells. (A) Chemical structure of osthole. (B) HGC-27 and SGC-7901 cells were treated with the indicated concentrations of osthole for 48 h, and the cell viability was measured by MTT assay. (C) HGC-27 and SGC-7901 cells were treated with or without osthole for 48 h and the cell morphology was determined under light microscopy (magnification, ×400). **P<0.01 vs. control.

To assess the anti-gastric cancer activity of osthole (Fig 1A), HGC-27 and SGC-7901 cells were treated with the indicated concentrations of osthole for 48 h, and the cell viability was examined using MTT assay. As shown in Fig 1B, osthole inhibited the cell viability of HGC27 and SGC-7901 cells in a dosage-dependent fashion. As the microscopy images in Fig 1C shown, after treated with 20, 40 or 80 μmol/l osthole for 48 h, the HGC27 and SGC-7901 cells exhibited obvious morphological features, such as shrinkage and distortion; besides, osthole treated cells became rounded, which was consistent with the MTT assay.

### Osthole induces cell cycle arrest at G2/M phase in gastric cancer cells

**Fig 2:**
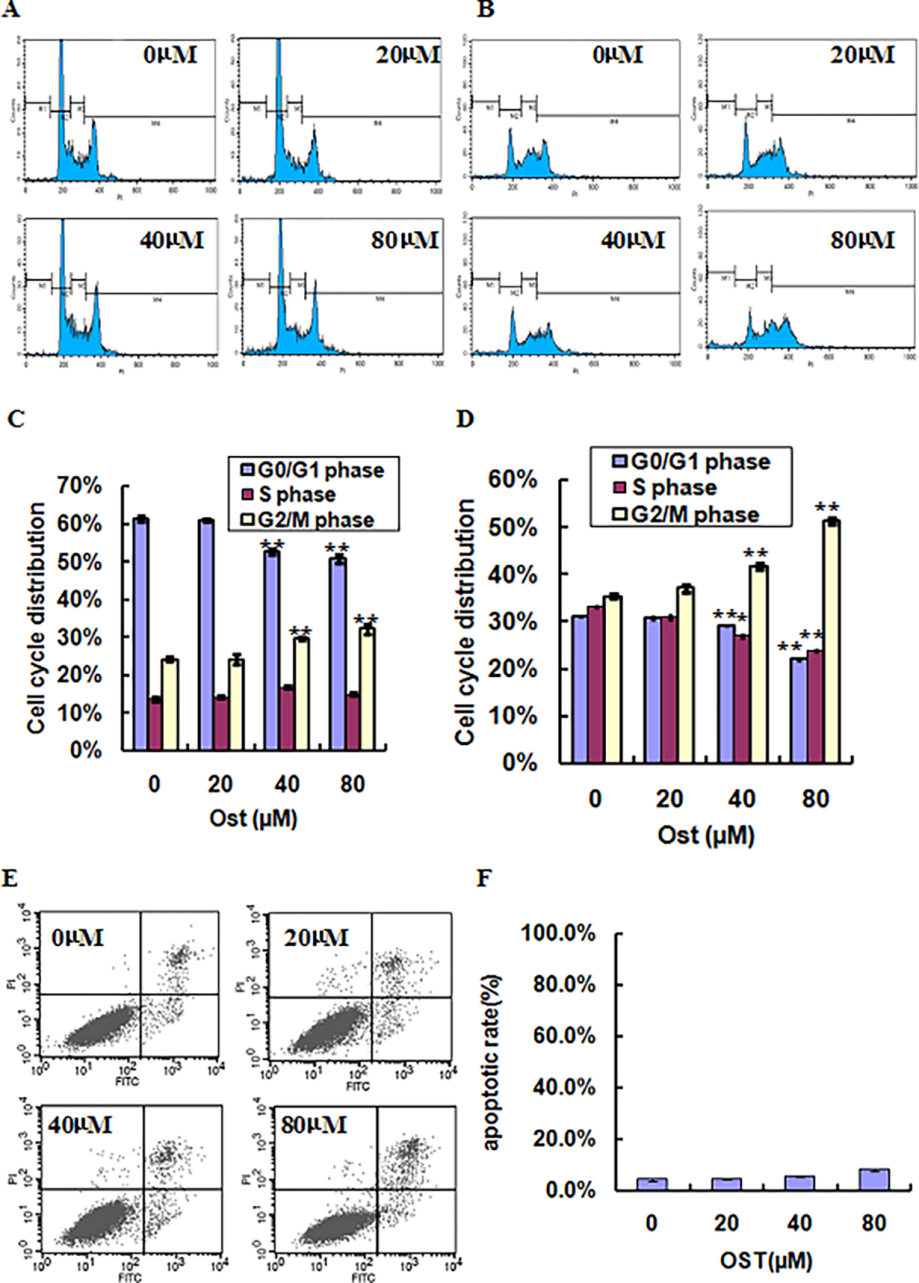
Osthole induces cell cycle arrest in gastric cancer cells. HGC-27 (A) and SGC-7901 (B) cells were treated with or without osthole for 48 h and the cell cycle proportions were determined by PI staining and flow cytometry. Quantitative data indicate cell cycle distribution of osthole-treated HGC-27 (C) and SGC-7901 (D) cells. HGC-27 cells were treated with or without osthole for 48 h and the cell apoptosis were assessed by Annexin V-PI staining (E). Quantitative data indicates cell apoptosis of osthole-treated HGC-27 cells (F). **P<0.01 vs. control.

To determine the possible mechanisms of osthole-mediated cell growth inhibition in HGC27 and SGC-7901 cells, cell cycle distribution was analyzed. HGC27 and SGC-7901 cells were treated with 20, 40 or 80 μmol/l osthole for 48 h and then the cell cycle phase was analyzed using PI staining and flow cytometry. As shown in Fig 2A and 2C, upon incubation with 20, 40 or 80 μmol/l osthole, the percentage of cells in G2/M phase were 24.2%, 29.7% and 32.2% after 48 h in HGC-27 cells, respectively, while the G0/G1 phase distribution was decreased between 61.0%, 52.8% and 50.8% when treated with 20, 40 or 80 μmol/l osthole, respectively. The similar cell cycle distribution pattern was also found in SGC-7901 cells in a concentration-dependent manner (Fig 2B and 2D). To further confirm whether osthole induced cell growth inhibition was associated with cell apoptosis, Annexin V-PI staining was conducted. As shown in Fig 2E and 2F, osthole treatment did not obviously induce gastric cell apoptosis at doses of 20, 40 or 80 μmol/l in HGC-27 cells, implying that osthole induced gastric cancer cell growth inhibition was not due to cell apoptosis. Collectively, these data revealed that osthole inhibits gastric cancer cell growth by induction of cell cycle arrest at G2/M phase.

### Osthole suppresses the expression of cyclinB1 and cdc2 proteins in gastric cancer cells

**Fig 3:**
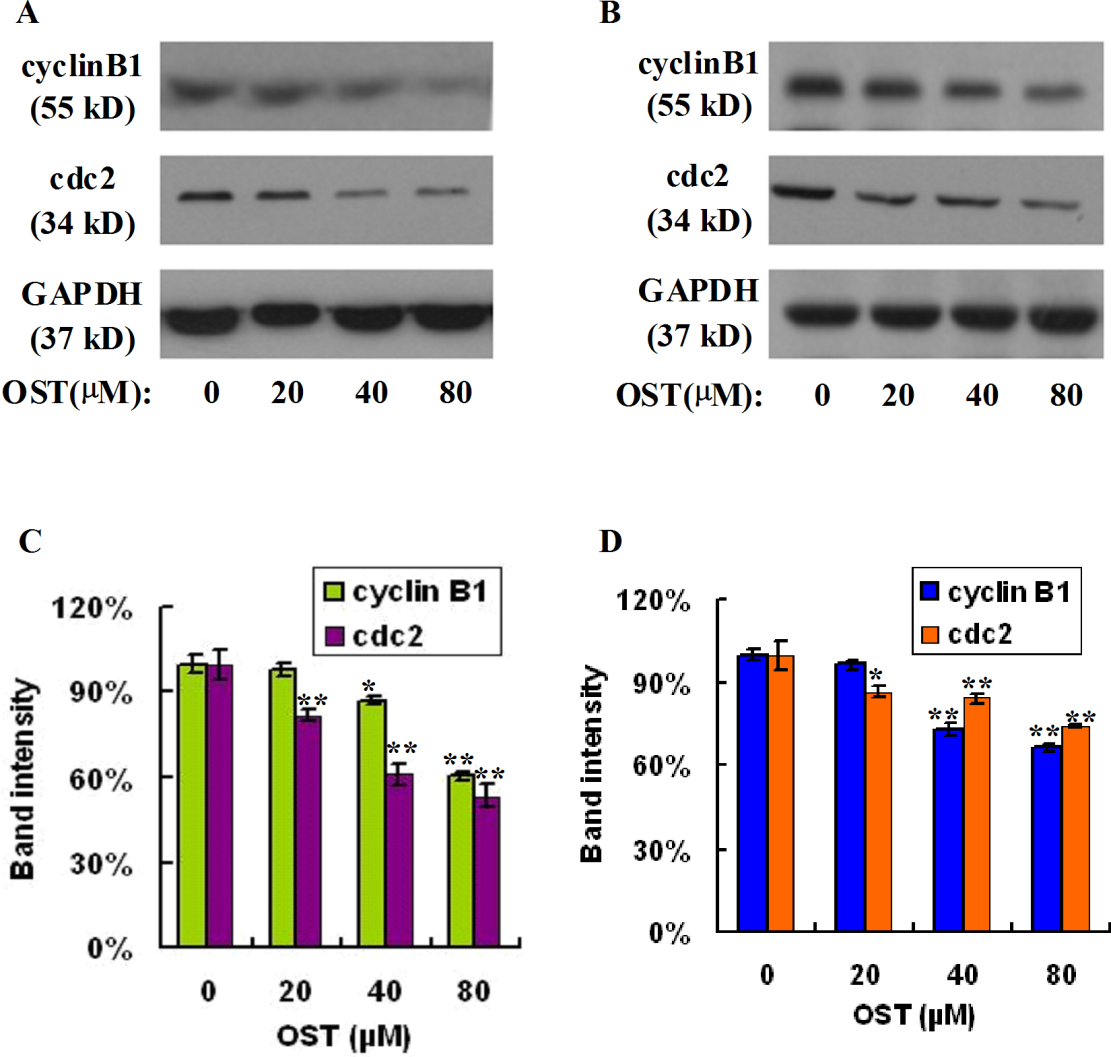
Osthole suppresses the expression of cyclinB1 and cdc2 proteins in gastric cancer cells. HGC-27 (A) and SGC-7901 (B) cells were treated with or without osthole for 48 h and subjected to sodium dodecyl sulfate-polyacrylamide gel electrophoresis and analyzed by western blot assay. The expression of cyclinB1 and cdc2 was measured by western blot assay. Densitometric analyses of cyclinB1 and cdc2 proteins were expressed as the mean ± SD from three independent experiments (C: HGC-27, D: SGC-7901). *P<0.05, **P<0.01 vs. control.

G2/M checkpoint could prevent DNA-damaged cells from entering mitosis, and repairing the DNA damaged in late S or G2 phases before mitosis. The cdc2 (Cdk1) -cyclinB complex is a key factor in the regulation of G2/M checkpoint [20]. In order to explore the molecular mechanisms of cell cycle arrest induced by osthole, we evaluated the effect of osthole on the expression of cyclinB1 and cdc2 proteins in HGC-27 and SGC-7901 gastric cancer cells. As shown in Fig 3A and 3C, following exposure to various doses of osthole in HGC-27 cells for 48h, the expression levels of cyclinB1 and cdc2 decreased significantly in a dosage-dependent fashion. Furthermore, a similar pattern was observed in another gastric cancer cells SGC-7901 (Fig 3B and 3D). The above results suggested that osthole induced cell cycle arrest at G2/M phase was associated with the decrease of cyclinB1 and cdc2 in gastric carcinoma cells.

### Osthole represses PI3K-AKT signaling in gastric cancer cells

**Fig 4:**
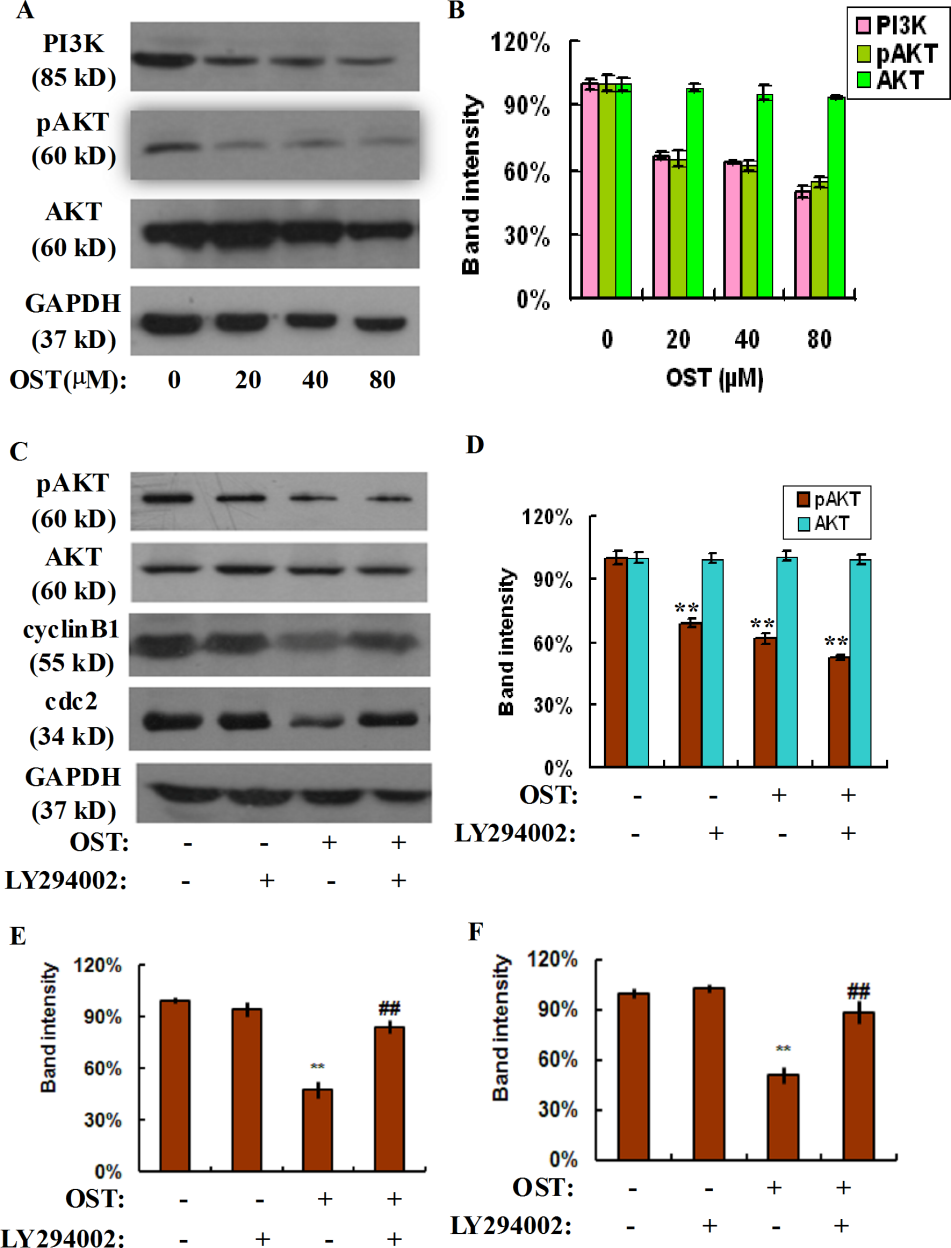
Osthole represses PI3K-AKT signaling in gastric cancer cells. HGC-27 cells were treated with or without osthole for 48 h and the cell lysates were subjected to sodium dodecyl sulfate-polyacrylamide gel electrophoresis and analyzed by western blot assay (A). The expression of PI3K, pAKT and AKT proteins was determined by western blot assay. Densitometric analyses of PI3K, pAKT and AKT proteins were expressed as the mean ± SD from three independent experiments (B). HGC-27 cells were pretreated with PI3K specific inhibitor LY294002 for 2 h and followed by the indicated concentration of osthole, the cell lysates were subjected to sodium dodecyl sulfate-polyacrylamide gel electrophoresis and analyzed by western blot assay. The expression of pAKT, AKT, cyclin B1, and cdc2 proteins was determined by western blot assay (C). Densitometric analyses of these proteins were expressed as the mean ± SD from three independent experiments (D: pAKT, AKT; E: cyclin B1; F: cdc2). **P<0.01 vs. control; ## P<0.01 vs osthole group.

The PI3K/Akt is one of the most important signaling pathways in controlling cell proliferation, cell cycle and apoptosis and plays a pivotal role in the development, progression and metastasis of various cancer cells [21]. In order to verify whether osthole mediated gastric cancer cells inhibition at G2/M phase was associated with PI3K/Akt signaling, we explored the expression of PI3K, pAkt and Akt after incubation with the different dosages of osthole for 48 h. As shown in Fig 4A and 4B, the levels of PI3K and its downstream target pAkt decreased significantly in a concentration-dependent manner exposure to osthole, while the total level of Akt was slightly changed in response to osthole, suggesting that PI3K/Akt signaling plays an important role in osthole mediated cell cycle arrest in gastric cancer cells.

To further confirm whether PI3K/Akt signaling was involved in osthole induced gastric cancer cell cycle arrest, we using a PI3K specific inhibitor, LY294002, in the following study. LY294002 pretreatment notably decreased the phosphorylation level of Akt compared with the osthole group in gastric cancer cells, while the total Akt level was unchanged (Fig 4C and 4D). Moreover, our western-blot data also showed that LY294002 pretreatment restored osthole induced down-regulation of cyclin B1 and cdc2 in human gastric cancer cells (Fig 4C, 4E and 4F). These results suggested that osthole induced cell cycle arrest at G2/M phase of gastric cancer cells by the modulation of the PI3K/Akt signaling.

## Discussion

It has been demonstrated that osthole, a coumarin derivative, possesses various pharmacological properties including immunomodulation, osteogenesis, neuroprotection and antioxidation and antitumor activities [9,6,8,7]. In terms of its anti-cancer property, osthole has been revealed to induce cell proliferation inhibition, apoptosis, cell cycle arrest and cell migration suppression in various tumor cells [17,16,10,15,11,12]. Nevertheless, no detailed studies have been demonstrated on its effect on gastric caricinoma cells still now. Our study aimed to elucidate the anti-gastric caricinoma activity of osthole and its underlying molecular mechanisms, and our data showed that osthole effectively inhibited cell growth and stimulated cell cycle arrest at G2/M phase in both gastric cancer HGC27 and SGC-7901 cells.

One of the major characteristics is the loss of control on cell proliferation, which develops partly due to the excessive regulation of cellular growth. The process of cell cycle depends on the strict control of cell cycle at different levels, and the core of these regulatory factors is consists of cyclins-dependent kinases (CDKs), cyclin and CDK inhibitors (CDKIs) [22]. As important regulators of the cell cycle machinery, CDKs are activated in normal cells and promote cell cycle progression from one to the next. Nevertheless, there are many changes in tumor cells, including CDK genes mutation, cyclins amplification and inactivation of CDKIs, which causes the dysfunction of CDK activity, mutation of cell cycle checkpoint, abnormal amplification of cell growth signals, resistance to cell apoptosis and so on [23]. Thus, searching for effective agents of cell cycle arrest induction has become one of the hotspots of cancer research. We evaluated the anti-gastric cancer effect of osthole on gastric cancer HGC27 and SGC-7901 cells using a MTT assay, and the results revealed that osthole effectively inhibited gastric cancer cell proliferation in a dosage-dependent pattern. Flow cytometric analysis indicated that osthole induced G2/M phase arrest in a dosage-dependent pattern, which might be associated with the down-regulation of cyclin B1 and cdc2. These data provided strong evidence in support of the notion that osthole has potent activity of anti-gastric carcinoma via cell cycle arrest and suppression of cyclin B1 and cdc2 *in vitro*.

Many documents have demonstrated that PI3K/Akt signaling pathway play an important role in the procession of cellular survival. PI3K/Akt signaling activates and promotes the phosphorylation of Akt cascade, which prevents oxidative damage. The regulation of osthole on PI3K/Akt signaling has been found in several cancers, including sarcoma [13], glioma [14], and lung cancer [24]. However, whether osthole suppresses gastric cancer cell growth was associated with PI3K/Akt signaling is not reported yet. Therefore, we evaluated the effect of osthole on PI3K/Akt signaling in gastric cancer cells. Our data showed that osthole effectively inhibited the expression of PI3K and p-Akt in gastric cancer cells, whereas the expression of total Akt is not obviously affected by osthole. Moreover, LY294002, a specific inhibitor of PI3K, has been used to further confirm the role of PI3K/Akt signaling in osthole mediated gastric cancer cells growth inhibition. The results showed that LY294002 pretreatment notably decreased the phosphorylation level of Akt compared with the osthole group in gastric cancer cells, and restored osthole induced down-regulation of cyclin B1 and cdc2 in human gastric cancer cells, implying that osthole may suppress gastric cancer cell cycle arrest via down-regulating PI3K/Akt signaling.

In summary, our data demonstrated that osthole exhibited a potential anti-gastric cancer property by inducing cell cycle arrest at G2/M phase. Moreover, our study also showed that PI3K/Akt signaling plays a key role in osthole mediated cell cycle arrest. Osthole could be a potential drug candidate for the treating of gastric cancer.
